# Effective inhibition of MERS-CoV infection by resveratrol

**DOI:** 10.1186/s12879-017-2253-8

**Published:** 2017-02-13

**Authors:** Shih-Chao Lin, Chi-Tang Ho, Wen-Ho Chuo, Shiming Li, Tony T. Wang, Chi-Chen Lin

**Affiliations:** 1 Ph.D. Program in Medical Biotechnology, National Chung Hsing University, Taichung, Taiwan; 2 Department of Food Science, Rutgers University, New Brunswick, New Jersey USA; 3 Department of Pharmacy, Tajen University, Pingtung, Taiwan; 4 Hubei Collaborative Innovation Center for the Characteristic Resources Exploitation of Dabie Mountains, Hubei Key Laboratory of Economic Forest Germplasm Improvement and Resources Comprehensive Utilization, Huanggang Normal University, Hubei, China; 5 Center for Infectious Diseases, Discovery Biology, SRI International, Harrisonburg, VA 22802 USA; 6 Department of Health and Nutrition, Asia University, Taichung, Taiwan; 7 Department of Medical Research, China Medical University Hospital, Taichung, Taiwan; 8 Department of Biomedical Sciences, National Chung Hsing University, 145 Xinda Rd., Taichung, 40227 Taiwan

**Keywords:** MERS-CoV, Middle East Respiratory Syndrome Virus, Resveratrol, MERS-CoV virus infection, Inhibition

## Abstract

**Background:**

Middle East Respiratory Syndrome coronavirus (MERS-CoV) is an emerging viral pathogen that causes severe morbidity and mortality. Up to date, there is no approved or licensed vaccine or antiviral medicines can be used to treat MERS-CoV-infected patients. Here, we analyzed the antiviral activities of resveratrol, a natural compound found in grape seeds and skin and in red wine, against MERS-CoV infection.

**Methods:**

We performed MTT and neutral red uptake assays to assess the survival rates of MERS-infected Vero E6 cells. In addition, quantitative PCR, western blotting, and immunofluorescent assays determined the intracellular viral RNA and protein expression. For viral productivity, we utilized plaque assays to confirm the antiviral properties of resveratrol against MERS-CoV.

**Results:**

Resveratrol significantly inhibited MERS-CoV infection and prolonged cellular survival after virus infection. We also found that the expression of nucleocapsid (N) protein essential for MERS-CoV replication was decreased after resveratrol treatment. Furthermore, resveratrol down-regulated the apoptosis induced by MERS-CoV *in vitro*. By consecutive administration of resveratrol, we were able to reduce the concentration of resveratrol while achieving inhibitory effectiveness against MERS-CoV.

**Conclusion:**

In this study, we first demonstrated that resveratrol is a potent anti-MERS agent *in vitro*. We perceive that resveratrol can be a potential antiviral agent against MERS-CoV infection in the near future.

## Background

Middle East Respiratory Syndrome (MERS) is a viral respiratory illness caused by a novel coronavirus (MERS-CoV) which was identified in Saudi Arabia in 2012 [1, 2]. Up to December 2016, the mortality rate of MERS patients is 35.4% with 652 deaths out of 1842 confirmed cases [3]. There is still no effective anti-MERS medicine or vaccine commercially available in the market. One previous study showed that stilbene derivatives could contain antiviral activities against Severe Acute Respiratory Syndrome Coronavirus (SARS-CoV) [4]. As a result, we tested whether a natural stilbene derivative, resveratrol (*trans*-3, 5, 4′-trihydroxystilbene) inhibits the MERS-CoV infection in this study. Resveratrol exists widely in different plants, including grape *(Vitis vinifera*), Huzhang (*Polygonum cuspidatum*) and cranberry (*Vaccinium macrocarpon*)[5]. In the past, resveratrol was demonstrated to decrease the production of nitric oxide in tissue, and thereby reduce inflammation [6–8]. Resveratrol also acts as an antioxidant to remove free radicals [9, 10], thus restrains tumor growth [11] and even age-related diseases [12, 13]. Resveratrol also inhibits STAT3 signaling pathway [14], the mTOR signaling [15], and the hedgehog signaling pathway [16]. Furthermore, resveratrol reportedly constrains infections caused by multiple pathogens, such as *Helicobacter pylori* [17], *Staphylococcus aureus* [18] or *Toxoplasma gondii* [19]. Interestingly, resveratrol has been demonstrated to exert antiviral effects against various viral infections, including Epstein-Barr virus (EBV) [20, 21], enterovirus 71 (EV71) [22], and herpes simplex virus (HSV) [23], as well as respiratory viral infections caused by influenza [24], respiratory syncytial virus (RSV) [25, 26], and rhinovirus [27]. However, it remains unknown whether resveratrol can inhibit MERS-CoV infection. In this study, we evaluated the antiviral effectiveness of resveratrol against MERS-CoV with an *in vitro* model.

## Methods

### Viral infection

Vero E6 cells (ATCC® Number: CRL-1586™) were planted on culture plates with 10% of fetal bovine serum (FBS) in DMEM for overnight before viral infection. MERS-CoV (HCoV-EMC/2012) was diluted to multiplicity of infection (M.O.I.) 0.1 with 2% FBS/DMEM and replaced the culture media in plates.

### MTT assay

MTT (3-(4,5-dimethylthiazol-2-yl)-2,5-diphenyltetrazolium bromide) can be reduced into formazan with purple color by cellular oxidoreductase as an indicator to access cell metabolism [28]. Briefly, Vero E6 cells were cultured on 96 well plates for overnight before administrating virus and resveratrol. After 48 hours incubation, supernatant was replaced with new culture medium and add 20 μL of 5 mg/mL MTT solution in each well. Plates were incubated at 37 °C for 1 hour before removing MTT-containing medium. Extract formazan with MTT solvent, which consists of 4 mM HCl and 0.1% Nondet P-40 (NP40) in isopropanol followed by measuring the level of purple formazan with ELISA reader (SpectraMax Plus 385, Molecular Devices®, USA) at wavelength 570 nm. The readouts obtained from MTT assay were further normalized to the value of uninfected cells where the value was set to 100%.

### Neural red uptake (NRU) assay

Cells were cultured on 96-well plate and infected by virus with or without treatment of resveratrol for 48 hours before performing NRU assay. The culture medium were removed and each well was added to 100 μL of 0.01% (w/v) neutral red in DMEM followed by incubating the plates at 37 °C, 5% CO_2_ for 1 hour. Neutral red/DMEM was removed and plates were washed twice with PBS. Neutral red dye was extracted with 100 μL/well of Sorensen Citrate Buffer and the plates was gently shaken for 5 min. The recovered neutral red dye was quantified by ELISA reader (SpectraMax Plus 385, Molecular Devices®, USA) at wavelength 540 nm. The results obtained were further normalized to the average readouts of tissue control groups where cell viability was set at 100%.

### Plaque assay

Vero E6 cells were planted in 12 well plates for overnight before conducting plaque assays. Viral samples were 10-fold serial diluted with MEM and added into cells. Cells were incubated with viral samples for one hour and rocked plates for every 15 min. After incubation, the inoculums were removed and cells were washed in PBS. MEM containing 1.5% agarose was then added to cells as overlay medium. Plates was incubated at 37 °C, 5% CO_2_ for 3 days after overlay medium was solidified and fixed directly with 0.2% crystal violet solution before counting plaques.

### Immunofluorescent assay

Vero E6 cells were seeded on 8-well chamber slides. Infected cells (treated with or without resveratrol) were first fixed in 4% paraformaldehyde for 15 minutes and then permeablized with 0.1% Triton X-100 for 10 mins. After 30 minutes blocking with 7.5% BSA at 37 °C, cells were immunostained with an anti-MERS-CoV N antibody (Sino Biological Inc., China) (1:500 dilution) at 4 °C overnight. After three washes with PBS, cells were then incubated with 1:1000 dilution of Alex Fluor® 568 anti-rabbit secondary antibody (Thermo Fisher, USA) for 1 hour. Cells were then washed three times in PBS with DAPI being added during the second wash. MERS nucleocapsid expression was examined by confocal microscope (LSM-700, Zeiss, Germany). For intracellular staining of MERS nucleocapsid protein, the protocol is similar to immunofluorescent assay for confocal microscope except the blocking buffer was 10% horse serum in 0.05% PBS-triton X100 and the secondary antibody was IRDye 800CW (Li-Cor®) with 1: 10,000 dilution in PBS.

### Quantitative real-time PCR

Total RNA samples of Vero E6 cells with or without MERS infection were isolated by RNeasy Mini kit (Qiagen®, Germany) according to the manufacturer’s instructions. Reverse transcription and PCR amplification were carried out with iTaq™ Universal One-Step RT-qPCR kit (Bio-rad®, USA) according to the manufacturer’s instructions. Real-time PCR was conducted by using StepOnePlus™ Real-Time PCR System (Appliedbiosystem®, USA) along with the following primer pairs: GAPDH-F: 5′-GAAGGTGAAGGTCGGAGTC-3′, GAPDH-R: 5′-GAAGATGGTGATGGGATTTC-3′ [29], MERS-CoV-F: 5′-CCACTACTCCCATTTCGTCAG MERS-CoV-R: 5′-CAGTATGTGTAGTGCGCATATAAGCA [30]. Each MERS RNA level defined as viral yield l was normalized with each GAPDH RNA level and relatively compared to MERS-CoV groups at 24 and 48 h.p.i. respectfully as relative RNA levels.

## Results

### Resveratrol reduced the cell death caused by MERS-CoV

**Fig. 1:**
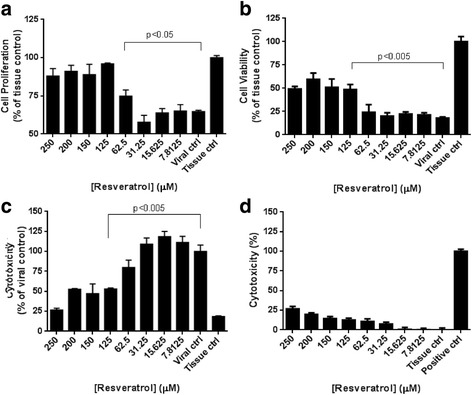
Resveratrol reduced the cell death caused by MERS-CoV infection. Vero E6 cells were infected by MERS-CoV with M.O.I of 0.1 and treated with resveratrol for 48 hours. The level of cell viability was determined by (**a**) MTS assay (**b**) neutral red uptake assay, and (**c**) LDH assay. **d** Resveratrol itself caused limited cytotoxicity to Vero cells by LDH assay

To investigate the anti-MERS-CoV effect of resveratrol, we directly treated MERS-CoV infected Vero E6 cells with different concentrations of resveratrol. Cells were infected with MERS-CoV at M.O.I. of 0.1. After 48 hours, we imaged the cellular morphology via microscopy and measured cell proliferation by MTT assay, cell viability by neutral red uptake (NRU) assay, and cytotoxicity levels by lactate dehydrogenase (LDH) assay. Resveratrol at 250 and 125 μM seems to alleviate the monolayer destruction of the Vero E6 cells infected by MERS-CoV (Fig. 1). Results from MTT assays (Fig. 1a) and NRU assays (Fig. 1b) showed that resveratrol can reduce the cell death induced by MERS-CoV infection in the concentration range from 250–125 μM. Also, resveratrol-treated groups revealed less cytotoxicity by LDH assay after MERS-CoV infection (Fig. 1c) and the cytotoxicity profiles correlated well with that of cell proliferation and cell viability assays. To determine if the observed antiviral effect by resveratrol was due to its cytotoxicity, we performed LDH assay for resveratrol treatment only. Figure 1d showed that resveratrol caused limited cytotoxicity to Vero E6 cells. Even at the highest concentration of 250 μM, the cytotoxicity was no more than 25%. In consistence, the cytotoxicity of MERS-CoV-infected cells was reduced by resveratrol treatment (250 μM) to approximately 25% (Fig. 1c). Therefore, we conclude that resveratrol reduced the cell death caused by MERS-CoV infection.

### Resveratrol reduced the RNA expression and viral yield of MERS-CoV

**Fig. 2:**
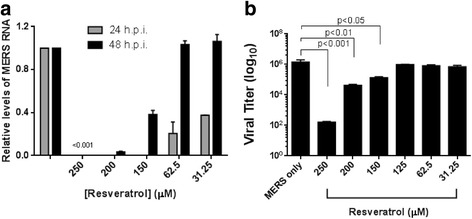
Resveratrol decreased MERS-CoV RNA and viral plaques. **a** MERS RNA level was monitored at 24 and 48 h.p.i. by real-time PCR after resveratrol treatment. Relative RNA levels were determined by comparing MERS only groups at each time point. GAPDH RNA was used as an internal control. **b** Quantification of plaque reduction assay of MERS-CoV titer after treated with resveratrol from 250 μM to 31.25 μM for 48 hours

To determine if resveratrol directly inhibits MERS-CoV infection, we assessed the effects of resveratrol on MERS-CoV viral production at the RNA level. We collected cell samples with or without viral infection after resveratrol treatments at 24 and 48 hours post-infection (h.p.i). Extracted total RNA was subjected to quantitative real-time PCR to compare the relative MERS-CoV RNA levels. Shown in Fig. 2a, the MERS-CoV RNA levels in resveratrol-treated cells at concentrations of 250, 200, 150, 62.5, and 31.25 μM were significantly lower than in MERS-CoV-infected cells at 24 h.p.i. However, the inhibitory effects of low concentrations of resveratrol, including 62.5 and 31.25 μM, diminished at 48 h.p.i. This data suggests that resveratrol treatment suppressed MERS-CoV RNA replication, although it requires relatively high concentrations of resveratrol to deliver persisted antiviral effects.

Next, we determined the infectious titer of MERS-CoV after resveratrol treatments by plaque reduction assays. Data in Fig. 2b showed that MERS titers were significantly reduced by resveratrol treatment at 250, 200 and 150 μM respectively, at 48 h.p.i. This result is consistent with those obtained from the quantitative real-time PCR at 48 h.p.i. In summary, these findings indicate that treatment of cells with resveratrol reduced the MERS-Cov RNA levels and infectious titers, which presumably accounted for the observed decrease in cell death.

### Resveratrol inhibited existing MERS-CoV infection

**Fig. 3:**
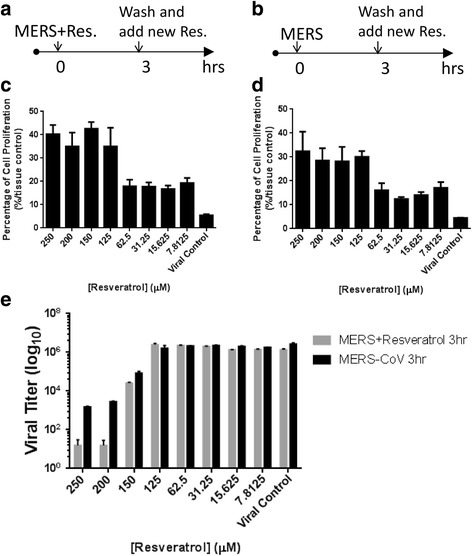
Resveratrol inhibited the existing MERS infection. Resveratrol and MERS-CoV were simultaneously added into cells for first 3 hours before removing virus and adding new resveratrol for the rest incubating time (**a**). MERS-CoV infected Vero cells for first 3 hours before washed out and treated with resveratrol (**b**). The trends of cell proliferation by MTT assays (**c** and **d**) and MERS-CoV titers by plaque assays (**e**) were similar, revealing resveratrol inhibited MERS viral yield even if existing MERS infection

Previous studies have shown that resveratrol exerted antiviral activities by blocking NF-κB pathway [22, 30], suggesting resveratrol has a broad spectrum of antiviral effects by down-regulating inflammatory signaling transduction. To determine whether resveratrol inhibits the entry or a post-entry step of MERS-Cov infection, we added resveratrol together with MERS-CoV immediately for 3 hours or after the infection has been initiated (Fig. 3a and b). We measured the cell proliferation (Fig. 3c and d) and determined the viral titers in the supernatants (Fig. 3e). The results demonstrated that even when resveratrol was given after MERS-CoV infection, it still reduced the viral titer. The same observations were made when cell proliferations and viral titers were measured, suggesting that resveratrol inhibits MERS-CoV infection after entry.

### Resveratrol inhibited MERS-CoV nucleocapsid expression

**Fig. 4:**
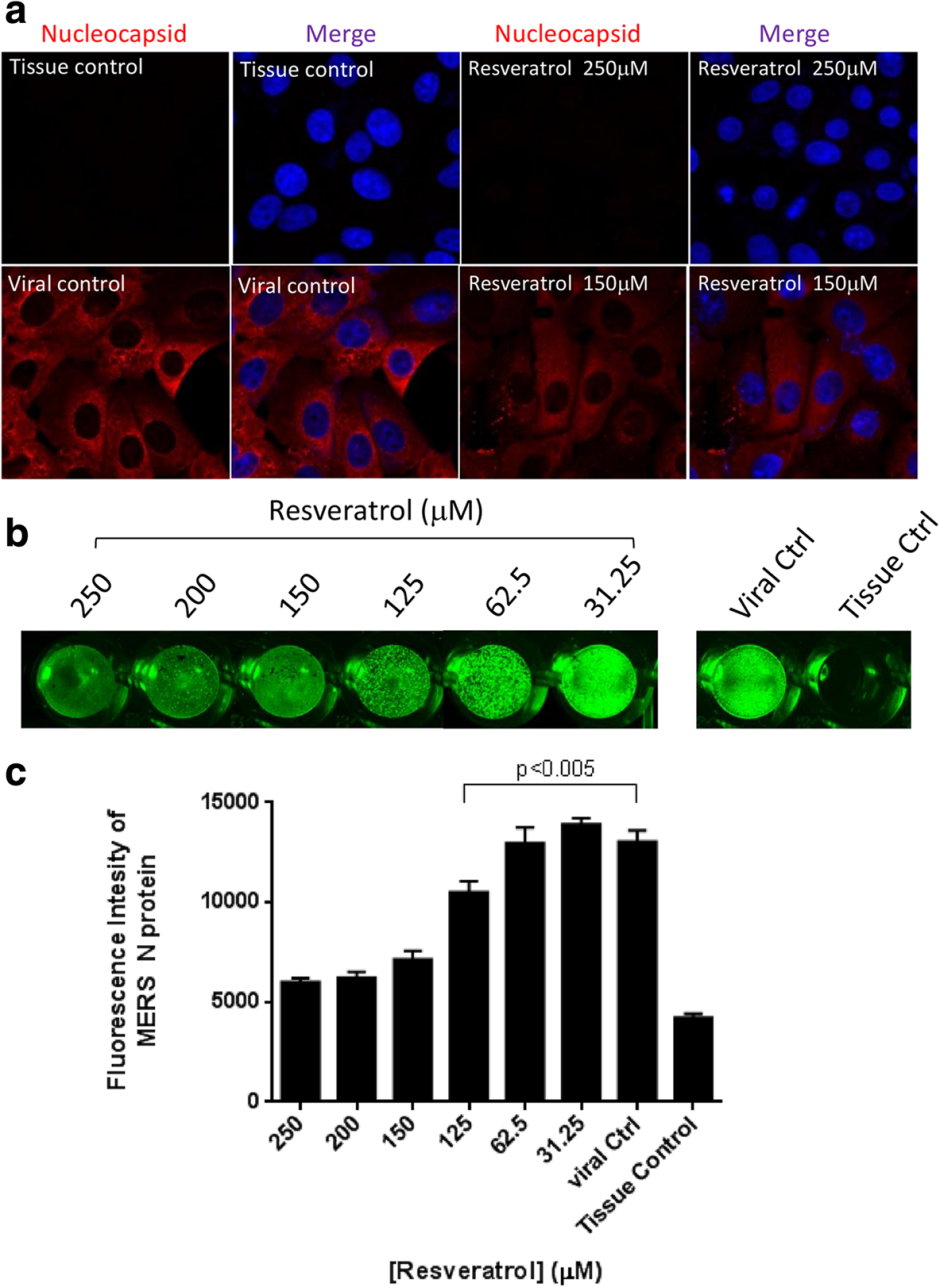
Resveratrol reduced nucleocapsid expression of MERS-CoV. Vero E6 cells were infected by MERS-CoV (M.O.I. 0.1) and treated with resveratrol for 24 hours followed by 4% paraformaldehyde fixation for immunofluorescent assays. **a** Nucleocapsid expressions were examined with confocal microscope at 680× magnification. DAPI was used for nucleus staining. **b** Intracellular staining of MERS nucleocapsid expressions were visualized by Odyssey® CLx Imaging system. **c** Quantification results of fluorescent intensities of MERS nucleocapsid proteins were determined by Odyssey® CLx Imaging software

To corroborate our findings, we stained nuclecapsid (N) protein of MERS-CoV after resveratrol treatments at 24 h.p.i and visualized the N protein distribution by confocal microscopy. Images shown in Fig. 4a demonstrated that 250 μM of resveratrol eliminated the N protein fluorescent signal compared to control groups, while 150 μM of resveratrol exhibited a limited decreasing of N protein signal. In order to elucidate whether the strength of N protein signal was correlated to the concentration of resveratrol, we performed intracellular staining of N protein in cells cultured in multiple well plates. MERS-infected Vero E6 cells were fixed and permeablized to facilitate anti-N primary antibody to access the target protein and quantified the strength of fluorescence by Li-Cor imaging system (Fig. 4b and c). The results showed that resveratrol remarkably inhibited MERS nucleocapsid protein translation in a dose-dependent manner, especially in the concentration of 250 to 125 μM.

### Resveratrol inhibited Caspase 3 cleavage induced by MERS-CoV infection

**Fig. 5:**
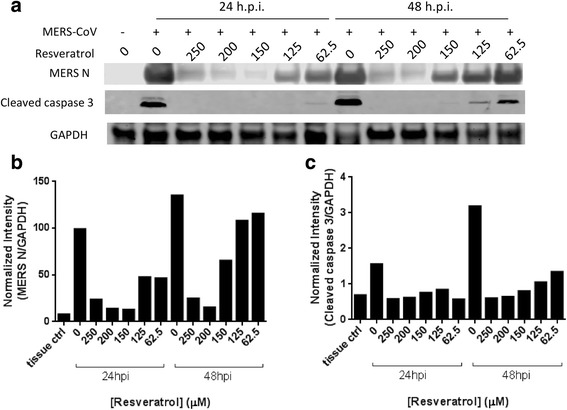
Resveratrol reduced MERS-induced cell apoptosis. Vero E6 cells were infected by MERS-CoV at M.O.I. of 0.1 and treated with resveratrol for 24 and 48 hours before collecting protein lysates. **a** Protein expression levels of MERS nucleocapsid and cleaved caspase 3 were evaluated by western blotting. GAPDH was used as a loading control. **b** and **c** Protein expression levels were quantified and then normalized with GAPDH expression. MERS nucleocapsid as well as cleaved Caspase 3 expressions were reduced by resveratrol in a dose-dependent manner

Apoptosis in different tissues during MERS-CoV infection has been widely documented [31, 32]. We therefore tested whether resveratrol is able to reduce the apoptosis induced by MERS-CoV. Due to the cleavage of Caspase 3, an indicator of apoptosis, was reportedly elevated during MERS-CoV infection [33], we collected the cell lysates after MERS-CoV infection and resveratrol treatments at 24 and 48 h.p.i. and conducted western blotting to measure the Caspase 3 cleavage levels. The results (Fig. 5) show that the protein expression of the cleaved Caspase 3 significantly increased after MERS-CoV infection, confirming that MERS-CoV could cause cellular apoptosis. Interestingly, when resveratrol was added, the levels of Caspase 3 cleavage decreased. As the concentration of resveratrol went higher, the levels of Caspase 3 cleavage decreased in a dose-dependent manner (Fig. 5c). Our results suggest that resveratrol reduced the MERS-CoV-mediated apoptosis. Notably, resveratrol at 250 μM did not lower the Caspase 3 cleavage to the same level, as did 200 μM (Fig. 5a, c). Given to the cytotoxicity of resveratrol itself, this result is expected.

### Consecutively administration of resveratrol at lower concentrations inhibited MERS infection

**Fig. 6:**
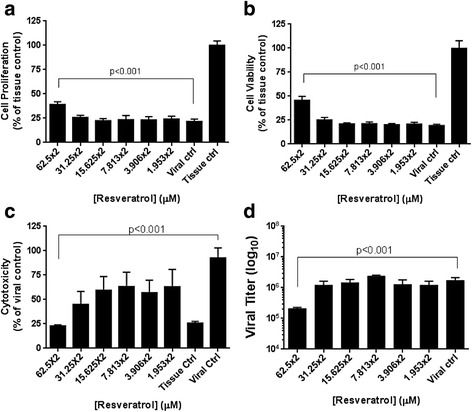
Consecutively resveratrol administration inhibited MERS infection in a lower dosage. MERS-infected Vero E6 cells were treated with resveratrol in lower dosages every 24 hours. The cell proliferation by MTT assay (**a**), cell viability by NRU assay (**b**), cytotoxicity by LDH assay (**c**), and plaque assay (**d**) were utilized to measure the cell survival after 48 hours of MERS infection at M.O.I. of 0.1

From the qPCR (Fig. 2a) and western blotting results (Fig. 5), we noticed that resveratrol at 62.5 μM and below appeared to inhibit MERS-CoV within 24 hours but then lost effects at 48 h.p.i. The loss of inhibitory effects at 48 hrs could due to the degradation of resveratrol in the media after such long period of incubation. Given the high dosages of resveratrol still has some cytotoxicity (Fig. 1e), lower dosages are more desirable to treat MERS-infected patients clinically. To explore the possibility of utilization of resveratrol at lower dosages, we added resveratrol consecutively (every 24 hrs) to MERS-infected cells at lower concentrations and evaluated the cell proliferation, cell viability, and cytotoxicity (Fig. 6). Interestingly, consecutive addition of resveratrol at 62.5 μM but not 31.25 μM or below partially rescued MERS-Cov-induced cell death (Fig. 6a-c) and lowered the production of infectious MERS-CoV by approximate tenfold (Fig. 6d), indicating resveratrol can inhibit MERS-CoV by administrating at lower dosages if given consecutively.

### Resveratrol exhibited extended antiviral activities

**Fig. 7:**
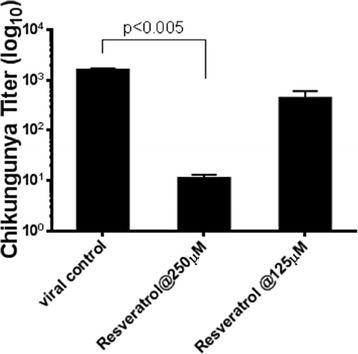
Resveratrol showed antiviral activity against chikungunya infection. Vero E6 cells were infected by chikungunya virus at M.O.I. of 0.1 and treated with resveratrol for 48 hours. The quantitative viral titers of chikungunya by plaque assay were decreased by resveratrol in 250 μM and 125 μM concentrations

Furthermore, we tested the effects of resveratrol on another emerging positive-sense RNA virus, chikungunya virus. As shown in Fig. 7, resveratrol not only inhibited MERS-CoV viral production but also reduced the production of chikungunya virus at concentrations of 250 and 125 μM. Altogether, our data suggests that resveratrol might be a lead candidate for further pre-clinical assessments of antiviral activity for MERS-CoV and additional emerging RNA viruses.

## Discussion

As an emerging human viral pathogen, MERS-CoV infection causes devastating diseases due to its high mortality. Therefore, it is urgent to develop vaccine or therapeutics. During the outbreak of Severe Acute Respiratory Syndrome (SARS) in 2002–2003, several antimicrobial agents were used to treat SARS-infected patients, including ribavirin, lopinavir/ritonavir, and type I interferon. However, most of the medication regimes showed no significant efficacy to SARS and came along with side effects, such as renal dysfunction or hemolytic anemia caused by ribavirin [34]. During the MERS-CoV epidemic, these treatments provided none or limited improvement in survival of patients and the efficacy remains unclear [35–37]. As a result, there is no effective remedial candidate during the MERS-CoV epidemic. In this study, we report the anti-MERS-CoV activities of resveratrol *in vitro*, providing evidence to support further detailed examinations of the potential clinical benefits for resveratrol in MERS-CoV infections.

We acknowledged that it is necessary to validate the anti-MERS efficacy of resveratrol *in vivo*. However, most of experimental animals, including rabbits, mice and ferrets, are asymptomatic after MERS infection [38–40]. Since the dipeptidyl peptidase 4 (DPP4) is a crucial receptor of MERS-CoV [41], we hypothesize that the human DPP4 expressed mice [42] could be a suitable model for examining the anti-MERS activity of resveratrol in the future.

Resveratrol itself has minor cytotoxicity even at high concentration of 250 μM, but it can be neglected comparing with the toxic levels caused by MERS-CoV infection, which were much greater than that of resveratrol itself. Considering the robust cell death caused by MERS-CoV, we think that resveratrol treatment remains a viable therapeutic strategy. In our study, we have shown that the resveratrol can be given either at high dosages up to 250 μM or at a relatively low concentration, such as 62.5 μM consecutively to treat MERS-CoV-infected cells.

In terms of the possible antiviral mechanisms for resveratrol, resveratrol has been reported to activate ERK1/2 signaling pathway [43] and promote cell proliferation and enhance SIR1 signaling [44], both of which are related to cellular survival and DNA repair in response to DNA damage [45, 46]. On the other hand, resveratrol could counteract the MERS-CoV-induced apoptosis by down-regulating FGF-2 signaling [47, 48]. In addition, MERS-CoV infection could lead to the production of inflammatory cytokines [49] whereas resveratrol may reduce the inflammation by interfering with the NF-κB pathway [50, 51]. In our study, the levels of cleaved caspase 3 were reduced by reseveratol after MERS-CoV infection. These changes may be results of direct inhibition of caspase 3 cleavage by resvertion of cell survival and the reduction of virus-induced apoptosis by resveratrol or inhibition of an upstream event that is required for caspase 3 cleavage. While the exact mechanism needs further investigation, the observed anti-MERS-CoV effect appears to be a collective result of the promotion of cell survival and the reduction of virus-induced apoptosis by resveratrol.

## Conclusion

In our study, we firmly found that resveratrol alone inhibits MERS-CoV infection. Future study will evaluate the potential synergy between resveratrol and other potential anti-MERS-CoV compounds to treat MERS-CoV infections.

## Abbreviations

ERK1/2: Extracellular signal-regulated protein kinases 1 and 2
LDH: Lactate dehydrogenase
MERS-CoV: Middle East Respiratory Syndrome Coronavirus
NF-κB: Nuclear Factor-kappa B
NRU: Neutral red uptake
SARS: Severe Acute Respiratory Syndrome
